# Aberrant Correlation Between the Default Mode and Salience Networks in Mild Traumatic Brain Injury

**DOI:** 10.3389/fncom.2020.00068

**Published:** 2020-07-28

**Authors:** Yongkang Liu, Wenzhong Wu, Xiao Chen, Minghua Wu, Gang Hu, Guoxing Zhou, Zhongqiu Wang, Rong Chen

**Affiliations:** ^1^Department of Radiology, Affiliated Hospital of Nanjing University of Chinese Medicine, Nanjing, China; ^2^Department of Acupuncture & Rehabilitation, Affiliated Hospital of Nanjing University of Chinese Medicine, Nanjing, China; ^3^Department of Brain Center, Affiliated Hospital of Nanjing University of Chinese Medicine, Nanjing, China; ^4^School of Pharmacy, Nanjing University of Chinese Medicine, Nanjing, China; ^5^Department of Radiology, Shanghai East Hospital, Tongji University School of Medicine, Shanghai, China; ^6^Department of Diagnostic Radiology and Nuclear Medicine, University of Maryland School of Medicine, Baltimore, MD, United States

**Keywords:** mild traumatic brain injury, intrinsic network, multilevel analysis, default mode network, salience network, network coupling

## Abstract

**Objectives:** The specific intrinsic network coupling abnormalities in mild traumatic brain injury (mTBI) patients are poorly understood. Our objective is to compare the correlations among the default mode, salience, and central executive networks in patients with mTBI and healthy controls.

**Methods:** This 2-year prospective study included 32 acute mTBI patients and 37 healthy comparisons. We calculated the functional connectivity scores among the default mode, salience, and central executive networks. Then we conducted multilevel correlation analysis to investigate component correlations, global graph, and local functional connectivity changes.

**Results:** Patients with mTBI showed significant increased functional connectivity between the anterior part of the default mode network and the salience network compared with controls (*p* = 0.013, false discovery rate correction). Hyper-connectivity between the default mode and salience network was significantly positively correlated with the dimensional change card sort score in patients with mTBI (*r* = 0.40, *p* = 0.037). The average path length of mTBI patients was significantly higher than that of controls (*p* = 0.028).

**Conclusions:** Aberrant functional coupling between the default mode and salience networks were identified in acute mTBI patients. Our finding has great potential to improve our understanding of the network architecture of mTBI.

## Key points

- Aberrant correlation between the default mode and salience networks in acute mTBI.
- Hyper-connectivity significantly positively correlated with the dimensional change card sort score.
- Understanding of the network architecture of mTBI.

## Introduction

Worldwide, traumatic brain injury affects about 10 million individuals annually (Hyder et al., 2007). Traumatic brain injury is associated with long-term disabilities including cognitive, psychological, motor, and sensory deficits. About 80% of traumatic brain injury patients are classified as mild traumatic brain injury (mTBI) (Kushner, 1998). Approximately 15% of mTBI patients have persistent neurological symptoms (Shenton et al., 2012).

fMRI is a non-invasive imaging technique for examining brain function. It uses changes in the BOLD signal to identify neuronal activity changes. Resting-state fMRI examines intrinsic functional connectivity in task-free conditions by mapping temporally synchronous, spatially distributed, spontaneous low-frequency BOLD signal fluctuation (Fox and Raichle, 2007). Resting-state fMRI provides a good signal to noise ratio and requires minimal patient compliance (Fox and Greicius, 2010). It has revealed a set of intrinsic connectivity networks. Voxels in an intrinsic connectivity network exhibit a coherent BOLD fluctuation pattern.

Many studies aimed at detecting changes in intrinsic connectivity networks in patients with traumatic brain injury (Sharp et al., 2014). Disruption of intrinsic connectivity networks could be a core mechanism of cognitive impairment in patients with traumatic brain injury. Resting-state fMRI studies have demonstrated complex patterns of intrinsic connectivity network abnormalities (Sharp et al., 2011; Shumskaya et al., 2012; Palacios et al., 2013; Pandit et al., 2013; Arenivas et al., 2014; Iraji et al., 2015). For example, Zhou et al. reported abnormal default mode network connectivity patterns in patients with mTBI which may provide insight into how neuronal communication and information integration are disrupted after mild head injury (Zhou et al., 2012).

The triple network model offers a theory for understanding cognitive dysfunction in brain disorders (Menon, 2011). The triple network model involves three intrinsic connectivity networks: the default mode network, the salience network, and the central executive network. The default mode network is anchored in the posterior cingulate cortex (PCC) and the medial prefrontal cortex (mPFC). It plays an important role in monitoring the internal mental landscape and is typically deactivated during most stimulus-driven tasks. The salience network is anchored in the dorsal anterior cingulated cortex (dACC) and frontoinsular cortex (FIC). It is involved in detecting, integrating, and filtering relevant interoceptive, autonomic, and emotional information. The central executive network is anchored in the dorsolateral prefrontal cortex (dlPFC) and posterior parietal cortex. It plays an important role in higher-order cognitive function and attention control. The triple network model states that the couplings among the default mode, salience, and central executive networks are responsible for the cognitive impairment in many brain disorders. This model has been examined in autism, schizophrenia, and frontotemporal dementia (Uddin, 2014).

Although many studies examined intrinsic connectivity network abnormalities in mTBI, no studies focused on the triple network model and examined correlations among the default mode, salience, and central executive networks in acute mTBI patients. Our study investigates correlations among the default mode, salience, and central executive networks in acute mTBI patients. We use multilevel correlation analysis, which examines functional connectivity changes across different scales, to analyze resting-state fMRI data. Understanding correlations among intrinsic connectivity networks holds great potential to improve our knowledge of the neuropathology of mTBI. Identifying neuroimaging features may lead to more accurate diagnosis and effective treatments.

## Materials and Methods

### Participants

From August 2012 to July 2014, mTBI patients and healthy comparisons were recruited in Shanghai Dongfang Hospital. mTBI patients were enrolled in the emergency department. For a comparison group, health subjects were recruited from the nearby community through advertisements. The hospital's institutional review board approved this study. All individuals provided written informed consent.

The diagnosis of mTBI was established based on the criteria of the American Congress of Rehabilitative Medicine for mild brain injury (American Congress of Rehabilitation Medicine, 1993). A subject was considered to have mTBI if any one of the following symptoms was evident following external application of force to the brain: (1) any period of loss of consciousness, (2) loss of memory for events immediately before or after the accident, (3) alteration in mental state at the time of the accident, or (4) focal neurologic deficits that may or may not be transient. The inclusion criteria were: (1) loss of consciousness of 30 min or less, (2) Glasgow Coma Scale (GCS) score of 13–15 at 30 min post-injury, and (3) duration of post-traumatic amnesia no longer than 24 h. The exclusion criteria were: (1) penetrating head injury, (2) uremia, liver cirrhosis, heart failure, pulmonary edema, coagulopathy, or renal dysfunction, (3) pregnancy, (4) *in vivo* magnetic implants (such as iron, cochlear implants, vascular clips, etc.) or pacemaker, (5) patient either died or had already received cardiopulmonary resuscitation before arrival at the hospital, (6) positive CT findings, (7) history of other neurological diseases, (8) history of neuropsychological diseases.

The comparison group included healthy subjects who had no history of neurological, psychiatric, or central nervous system disease, and no prior TBI. All participants (mTBI and healthy subjects) were right-handed. Healthy subjects were matched to mTBI subjects by age, sex, education, and handedness at the group level.

Among 71 participants, two subjects with significant MRI motion problems were excluded. The final dataset included 32 acute stage mTBI patients and 37 healthy comparisons.

### MRI Protocol

MR imaging was performed in mTBI patients within 7 days of the injury. MR data were acquired with a Philips Achieva 3.0T TX MRI scanner (Royal Philips, Amsterdam, Netherlands). The MR protocol included anatomical imaging (T1 and T2), resting-state fMRI, and DTI. High-resolution T1-weighted structural images were acquired with a MPRAGE sequence. The imaging parameters were TR/TE = 8.2/3.5 ms; flip angle = 8 degree; slice thickness = 1 mm, voxel size = 1 × 1 mm, FOV = 256 × 256. The T1 acquisition time was 4.56 min. Resting-state fMRI data were acquired with a FE-EPI sequence. The sequence parameters were: TR/TE = 1,500/35 ms, flip angle = 90 degree, slice thickness = 5 mm, voxel size = 3.75 × 3.75 mm, FOV = 240 × 240 mm, acquired matrix = 64 × 64, dyn = 210. Participants were asked to keep their eyes focused on cross-hairs projected onto a screen, and not think of anything during image acquisition. The total resting-state fMRI acquisition time was 5.35 min. Diffusion tensor images were acquired with a single-shot echo-planar sequence (TR/TE = 9,000/90 ms, slice thickness = 2 mm, voxel size = 2 × 2 mm, field of view = 256 × 256 mm). Diffusion gradients were set in 32 non-collinear directions by using two *b*-values (*b* = 0 and 1,000 s/mm^2^). The total DTI acquisition time was 5.5 min.

### Neuropsychological Assessment

Neuropsychological tests were administrated within 24 h of MR imaging. We used the Dimensional Change Card Sort test (DCCS) to assess executive functioning (Zelazo, 2006). The DCCS is a standard procedure for measuring executive function, specifically tapping cognitive flexibility. In the DCCS, two target pictures are presented that vary along two dimensions (e.g., shape and color). Participants match a series of bivalent test pictures to the target pictures, first according to one dimension (e.g., color) and then, after several trials, sort the same cards a new way (e.g., shape). In “switch” trials, the participant must change the dimension being matched. Scoring is based on a combination of accuracy and reaction time. This combination score is converted to a scale score with mean 100 and standard deviation (SD) 15. Higher scores indicate higher levels of executive functioning. DCCS was chosen because subjects with TBI often suffer impairments in their cognitive flexibility as a result of brain damage (Whiting et al., 2017).

### Multilevel Correlation Analysis

**Figure 1:**
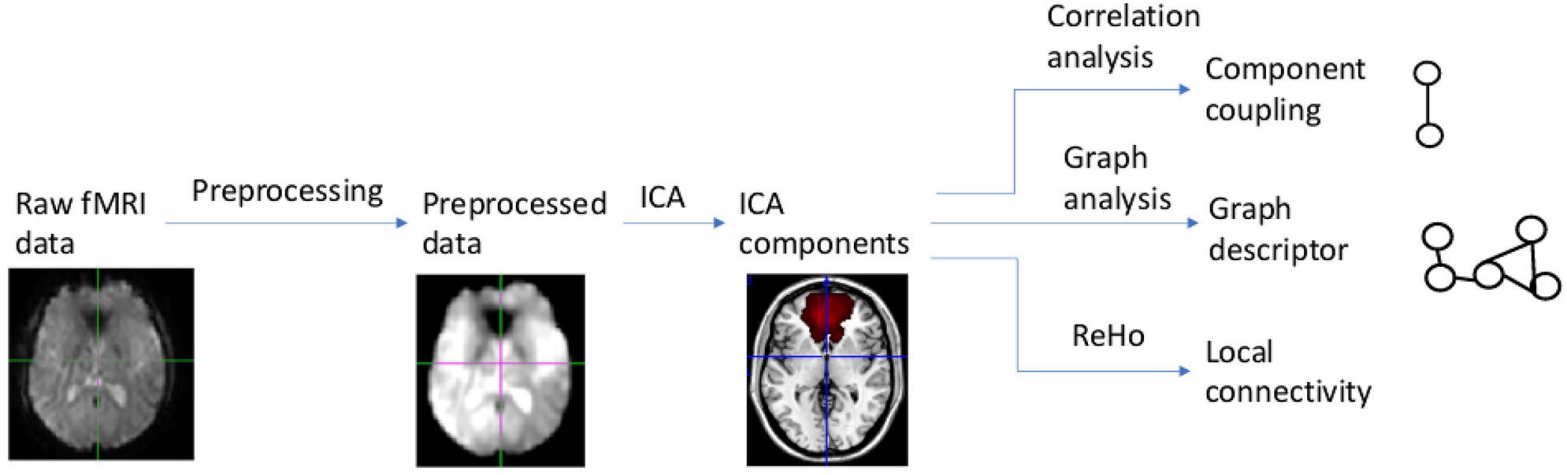
Diagram of multilevel correlation analysis.

Our data analysis and modeling method, called multilevel correlation analysis, is summarized in Figure 1. Multilevel correlation analysis centers on modeling correlations among the default mode, salience, and central executive networks. It includes the following modules: preprocessing, independent component analysis, component correlation analysis, graph analysis, and local connectivity analysis. Multilevel correlation analysis examines connectivity changes across different scales. Component correlation analysis focuses on localized changes in correlations among components. Graph analysis examines distributed, global level changes in information communication among components. Local connectivity analysis probes local functional connectivity changes.

### Image Preprocessing

Our resting-state fMRI data preprocessing pipeline was based on FMRIB Software Library (FSL) (Jenkinson et al., 2012) and Analysis of Functional NeuroImages (AFNI) (Cox, 1996). This pipeline (Chen et al., 2016) included the following steps: skull stripping, slice-timing, and motion correction, Gaussian spatial smoothing, temporal filtering, regressing out nuisance parameters, and spatial normalization to the Montreal Neurological Institute (MNI) space.

We first dropped the first ten volumes to remove T1 equilibration effects. Then we performed slice-timing and motion correction followed by skull-stripping. Skull-stripping results were verified by visual inspection. We smoothed fMRI volumes using a Gaussian kernel with a full width at half maximum = 6 mm, and temporally filtered fMRI volumes with bandwidth = [0.005Hz 0.1Hz]. We extracted a base volume from the 4D fMRI volume and registered this base volume to the subject's T1 volume using the mutual information-based registration in FSL. Based on the subject's T1 image segmentation results, we obtained white matter and CSF masks. We calculated the mean white matter and CSF signals. Then we regressed out the six motion parameters, the mean frame-wise displacement (FD), and the mean white matter and CSF signals. We registered the preprocessed 4D volume to the MNI space by applying the composite deformation field which combined the deformation field from the base volume to the subject's T1 volume, and that from the subject's T1 volume to the MNI space.

Excessive head motion is known to induce artifacts and false-positive correlations among brain structures in resting-state fMRI (Power et al., 2012). We employed relatively strict criteria to address the head motion problem. First, we excluded all subjects with mean FD of more than two standard deviations above the sample mean. Second, we regressed out head-motion parameters and mean FD from the BOLD signal.

### Independent Component Analysis

We used Independent Component Analysis (ICA) to identify intrinsic networks. We performed probabilistic ICA (Beckmann and Smith, 2004) by applying MELODIC (Multivariate Exploratory Linear Optimized Decomposition into Independent Components) implemented in FSL to the preprocessed resting-state fMRI data from the comparison group. A multi-session temporal concatenation tool in MELODIC was used; variance normalization also was used. We detected component masks based on comparison group data for two reasons: first, applying ICA to data from both the mTBI and comparison groups is less sensitive in detecting differences in network changes (Rytty et al., 2013). Second, ICA results based on the comparison group are a more robust match with previous healthy subject group ICA templates (Rytty et al., 2013). The number of components used was 30 because previous studies found ICA with 30 components can reliably identify intrinsic networks (Shumskaya et al., 2012). MELODIC converted the estimated ICA maps to *Z* statistic images using a mixture model approach. The *Z* statistic images were thresholded with *Z* = 4 (Beckmann et al., 2009). Based on visual inspection of spatial maps and the related time courses and power spectrums, we identified components as ventricular, vascular, susceptibility, or motion-related artifacts (Kelly et al., 2010); these noise components were excluded from the analysis.

For an ICA component spatial map, we determined to which intrinsic network it belonged by using template matching. We used the Allen intrinsic network template (Allen et al., 2011) as the intrinsic network template because it is widely used to examine brain networks in health and disease. We calculated the cross-correlation between a component and a reference network and determined to which reference network the component belonged. We selected ICA components that were in the default mode, salience, and central executive networks. Subject-specific time courses relating to ICA component maps were extracted using dual regression. For each subject, the group-average set of spatial maps was regressed into the subject's 4D volumes. This resulted in a set of subject-specific time courses for ICA component spatial maps.

### Component Correlation Analysis

Component correlation analysis centers on studying correlations among components. Each ICA component constituted a node. For each subject, we calculated the node time course; then calculated the Pearson correlation coefficient between a time course pair. We converted it to a *Z* score using Fisher's *Z* transformation. This *Z* score was referred to as a functional connectivity score. For a study with *K* components, we generated a *K* × *K* functional connectivity matrix for each subject.

For a functional connectivity score, we used the Wilcoxon rank-sum test to determine whether there was a significant difference between mTBI patients and healthy subjects. We used the False Discovery Rate (FDR) to address multiple comparisons. If the FDR corrected *p*-value was smaller than 0.05, there was a significant difference across groups. Such a score was a feature characterizing mTBI. Let **F** denote the detected feature set.

To assess the stability of our findings, we conducted a Jackknife resampling-based analysis. We removed one sample from the original dataset and then conducted the analysis based on the remaining samples. This resulted in a resampling based feature set. If our dataset has *n* samples, we will generate *n* resampling-based feature sets. We compared the original feature set with resampling based feature sets and calculated a stability metric λ. The stability metric was defined as *N*_[original=resampling]_/*n*, where *N*_[original=resampling]_ was the number of times that the original feature set was the same resampling based feature set, and *n* was the sample size. λ was between 0 and 1. Greater λ represented a more stable model.

### Association With Executive Function

To investigate brain-behavior associations, we performed two association analyses. First, we conducted a correlation analysis between the detected feature and the DCCS score for mTBI subjects. Second, for all subjects (healthy and mTBI subjects), we conducted a regression analysis with the DCCS score as the dependent variable and the detected feature and the group-membership variables as predictors. This analysis detected group differences in the association between the DCCS score and the detected imaging feature. Normality was checked by the Shapiro-Wilk test.

### Graph Analysis

Graph analysis uses graph theoretical methods to analyze functional connectivity matrices. Relative to component correlation analysis, it can generate global graph descriptors to characterize the topological or information-theoretical complexity of a graph. Graph analysis and component correlation analysis provide complementary information about couplings among the default mode, salience, and central executive networks.

An ICA component in the default mode, salience, and central executive networks was a node in a graph. We calculated the Pearson correlation coefficient between a component-time course pair; and converted it to a *Z* score. We generated a connectivity matrix for each subject. To generate a graph, we thresholded the connectivity matrix based on graph density. The density threshold was chosen as 0.35 < graph density < 0.4 (step size was 0.02). The lower limit was chosen to prevent a disconnected graph; and the upper limit was chosen because brain networks in general are not densely connected and have a density <0.4 (Sporns, 2011). The thresholding step generated a weighted graph. Then we calculated two graph descriptors: the global clustering coefficient and average path length (Rubinov and Sporns, 2010). The clustering coefficient of a node was defined as the likelihood of the neighborhoods being connected with each other. The global clustering coefficient was the average of the clustering coefficient over all the nodes. The path length between the nodes was the sum of the edge length along the path. The average path length was the average of the shortest path length across node pairs. Both the global clustering coefficient and average path length were calculated based on the weighted graph. For each threshold, we calculated a graph descriptor. An aggregated graph descriptor was generated by calculating the area under the curve (AUC) across graph density thresholds (Bullmore and Bassett, 2011).

### Stability Relative to ICA Component Mask Generation

We examined whether our findings were sensitive to the method generating ICA component masks. Let Mask(control) denote that ICA masks are generated based on controls. Let Mask(all) denote that ICA masks are generated based on all subjects (controls and mTBI). We rerun the whole ICA workflow using Mask(all). We used fslcc (Jenkinson et al., 2012) to match Mask(control) and Mask(all). If a component in Mask(controls) matched multiple components in Mask(all), we merged these component masks in Mask(all). With this transformation, we can compare results in the Mask(control) and Mask(all) spaces.

### Local Functional Connectivity Analysis

Local functional connectivity quantifies local functional couplings among spatially adjacent voxels. Regional homogeneity (ReHo) is widely used to examine local functional connectivity (Zang et al., 2004). In ReHo, Kendell's coefficient of concordance was used to measure regional homogeneity or similarity of the ranked time series of a given voxel with its nearest 26 neighbor voxels in a voxel-wise way. The intracranial voxels were extracted to generate a mask. Then 3D ReHo in AFNI was used to generate the ReHo map. Each subject's ReHo map was divided by its own mean ReHo within the brain mask for standardization purposes (Zang et al., 2004). Then voxel-wise *t*-test analysis was performed to detect voxels whose ReHo values were significantly different across groups. Monte Carlo simulation in AFNI was used for multiple comparison correction. Voxels with corrected *p*-value < 0.05 were significantly different across groups.

### Anatomical Connectivity Analysis

To investigate anatomical connectivity underlying functional connectivity, we used Tract-Based Spatial Statistics (TBSS) implemented in FSL to detect changes in brain anatomical connectivity. We analyzed fractional anisotropy (FA) which quantifies local tract directionality and integrity. TBSS projects a subject's FA map to a common space, creates an FA skeleton, and projects each subject's FA onto the skeleton to generate a skeletonized FA image for each subject. This skeletonized FA image represents brain anatomical connectivity. The white matter integrity differences were investigated by using the threshold-free cluster enhancement at *p*-value < 0.05 (5,000 permutations) fully corrected for multiple comparisons. If a voxel's corrected *p*-value was smaller than 0.05, we considered this voxel's FA changed across groups.

We also performed ROI based anatomical connectivity analysis. ROIs were defined using the Johns Hopkins University white matter tractography atlas. There was a total of 20 ROIs in our analysis representing major white matter fiber tracts. For each ROI, we calculated the average FA value in the skeletonized FA map. Then we used independent two-group Mann-Whitney *U*-test to identify ROIs whose FA values were different between the mTBI and comparison subjects. The FDR correction was used to correct for multiple testing.

## Results

### Participant Characteristics

Participant characteristics are summarized in Table 1. Mean ages were 30 years (SD 6.0) and 31 years (SD 8.7) for mTBI patients and comparisons, respectively. There were no significant difference in age across groups (*p*-value = 0.37, two-sample *t*-test); no significant difference in the female:male ratio (*p*-value = 0.81, chi-square test); and no significant difference in education (*p*-value = 0.11, two-sample *t*-test). All subjects were right-handed.

**Table 1:** Demographic (age, sex, education), neurocognition (DCCS), and disease severity (Rivermead post-concussion questionnaire and brief symptom inventory-18) of participants.

	**mTBI (*n* = 32)**	**Controls (*n* = 37)**	***P***
	**Mean (SD)**	**Mean (SD)**	
Age (years)	30 (6.0)	31 (8.7)	0.37
Sex (female/male)	12/20	16/21	0.81
Education (years)	15 (2.2)	16 (2.2)	0.11
Dimensional change card sort test	98 (12.8)	103 (10.9)	0.01
Glasgow coma scale	15 (0.2)	−	−
Rivermead post-concussion questionnaire	16 (13.2)	−	−
Brief symptom inventory-18	15 (14.4)	−	−

*SD, standard deviation*.

For mTBI patients, the cause of injury included traffic accidents (Nine patients), falls (Two patients), sports-related accidents (Two patients), and objects striking the head (19 patients). The injury severity in mTBI patients was mild: 97% with GCS = 15. The RPQ measures severity of 16 post-concussion syndrome symptoms, as compared to the premorbid level. The mean RPQ was 16 (SD 13.2). The BSI-18 is a tool to assess the level of psychological distress after mTBI. It covers somatization, depression, and anxiety. The mean BSI-18 was 15 (SD 14.4).

T1- and T2-weighted images were reviewed by an experienced neuroradiologist to identify structural abnormalities, including assessment for evidence of hemorrhage. The neuroradiologist was blinded to the group membership and clinical information. The images were found to be free of structural abnormalities for both mTBI patients and comparisons.

### Component Correlations

**Figure 2:**
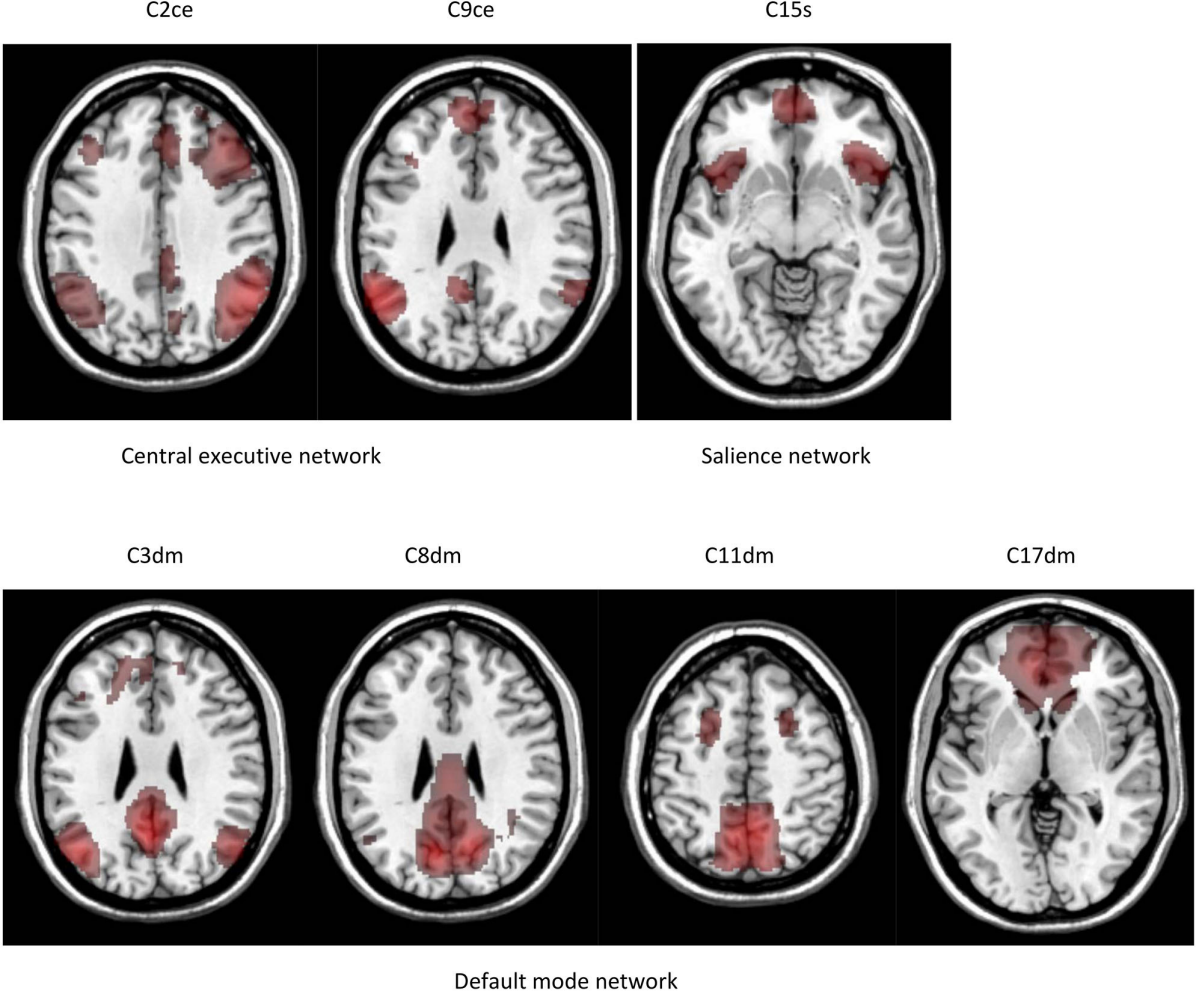
Intrinsic connectivity networks identified by group ICA. The results are in the Mask(control) space.

We performed a 30-component group ICA using resting-state fMRI data from 37 comparison subjects. The component masks were in the Mask(control) space. Five components were identified as noise or artifact components. The remaining components were intrinsic connectivity networks. Our study centered on seven components in the default mode, salience, and central executive networks. These components are depicted in Figure 2. Components 2 and 9 are in the central executive network; components 3, 8, 11, 17 are in the default mode network; and component 15 is in the salience network. The central executive network includes two components. Component 2 is in the dlPFC and posterior parietal cortex, and component 9 is in the posterior parietal cortex. The default mode network includes four components. Components 3, 8, 11 are in the posterior part of the default mode network including the PCC and precuneus. Component 17 is in the anterior part of the default mode network anchored in the mPFC. The salience network is represented by component 15 which is primary in the dACC and the anterior insular.

**Figure 3:**
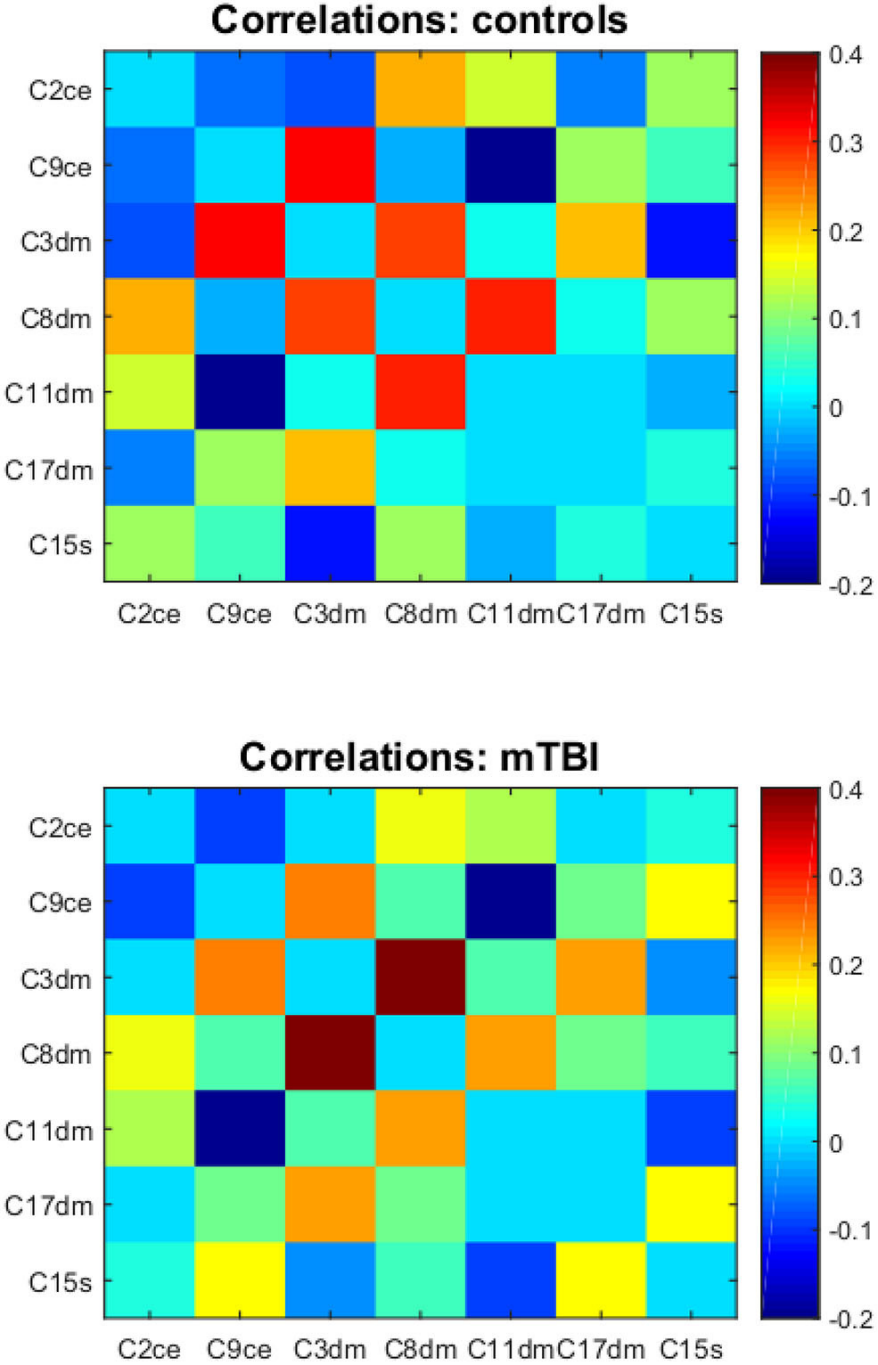
The component correlation analysis results in the Mask(control) space. Top and bottom are the mean functional connectivity correlation matrices for controls and mTBI subjects, respectively. C2ce, C9ce—the central executive network; C3dm, C8dm, C11dm, C17dm—the default mode network; C15s—the salience network.

Functional connectivity matrices for comparisons and mTBI patients are depicted in Figure 3. Correlations between component time courses revealed connectivity patterns consistent with known functional relationships. We observed a positive correlation between components in the same intrinsic network. We also found negative correlations between the default mode network components and components in the salience and central executive networks.

**Figure 4:**
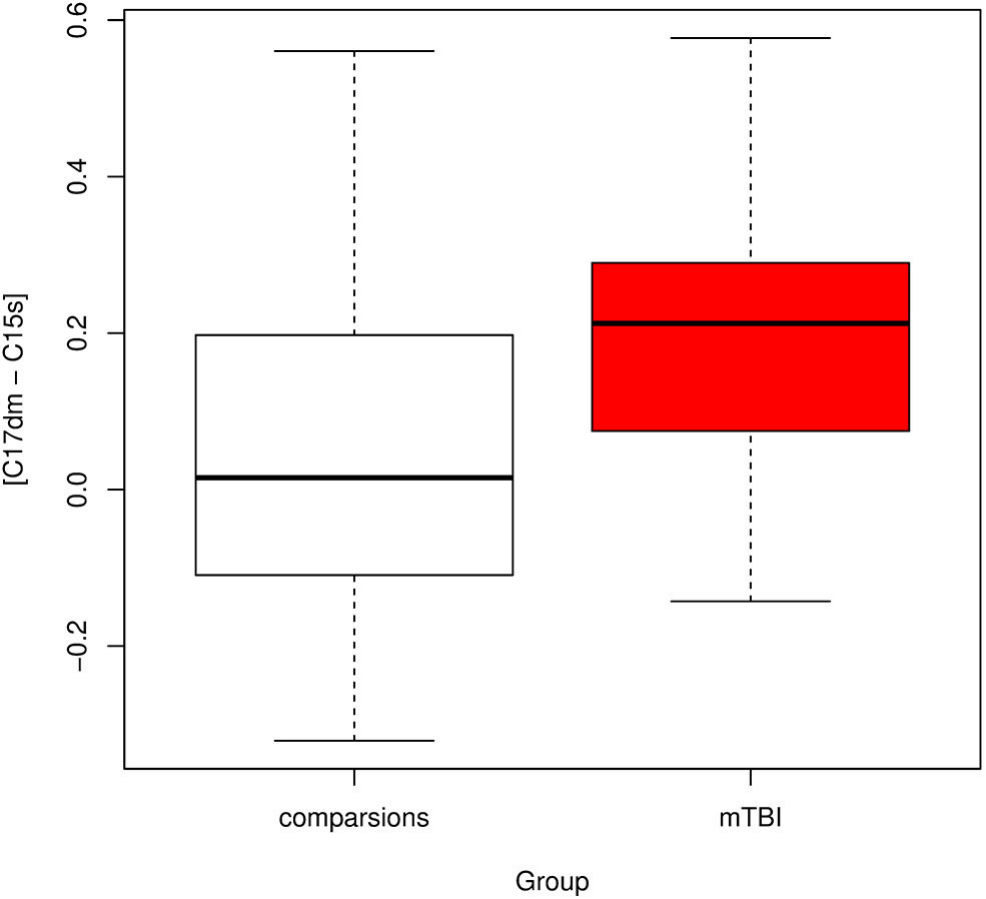
Boxplot of connectivity between components 17 and 15 [C17dm – C15s]. C17dm is in the default mode network and C15s is in the salience network.

The correlation between a component in the default mode network (component 17) and a component in the salience network (component 15) was significantly different between mTBI and healthy subjects with corrected *p*-value = 0.013 (Figure 4). Let [C17dm – C15s] denote the connection between component 17 (part of the default mode network) and 15 (part of the salience network). The mean functional connectivity scores of [C17dm – C15s] were 0.037 (SD 0.22) and 0.201 (SD 0.18) for comparisons and mTBI patients, respectively.

We compared the resampling based feature sets to the original feature set and found that the original feature set was remarkably stable. For all 69 resampling based feature sets, we detected [C17dm – C15s] were significantly higher in mTBI patients than comparison subjects (corrected *p*-value < 0.05).

### Association With Executive Function

**Figure 5:**
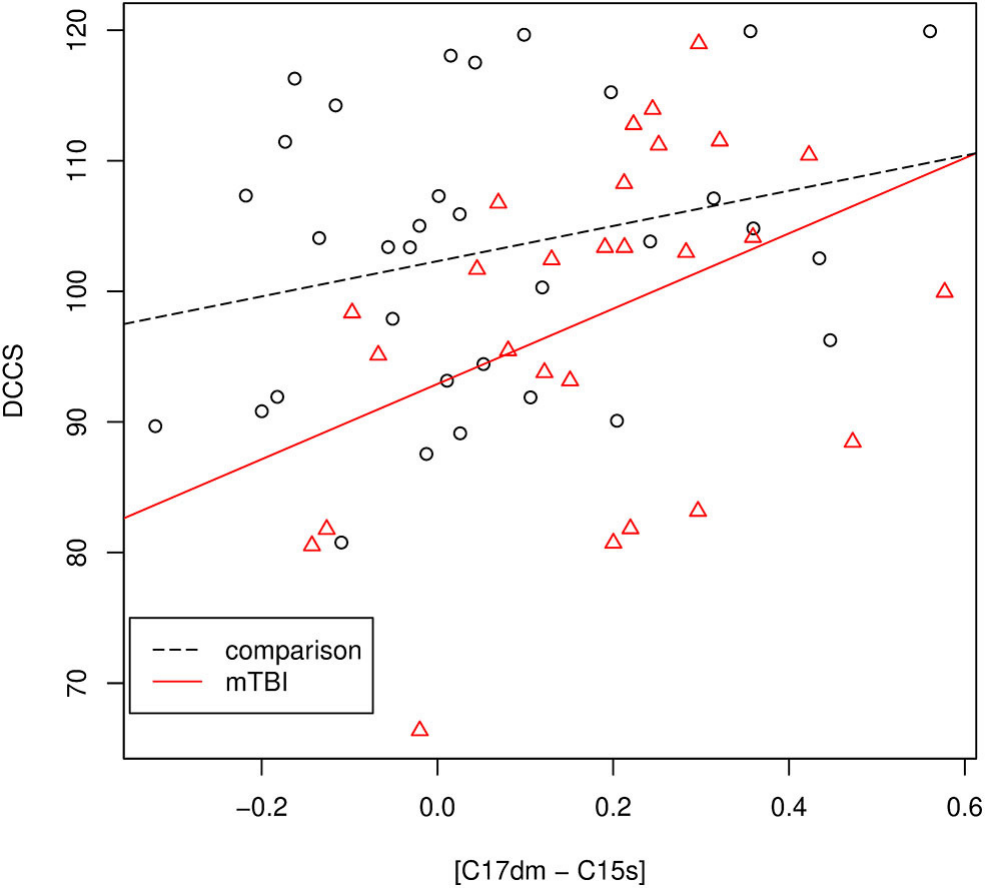
The association between the DCCS score and [C17dm – C15s]. Data points with circle shape are controls and data points with triangle shape are mTBI patients.

The results of the association analysis are depicted in Figure 5. For mTBI patients, we performed Pearson's correlation analysis between the DCCS score and [C17dm – C15s]. The DCCS score was positively associated with [C17dm – C15s]. The correlation coefficient was 0.40 (*p*-value = 0.037). To assess whether the association between DCCS and [C17dm – C15s] was related to mTBI severity, we built a regression model with DCCS as the dependent variable and [C17dm – C15s] and mTBI severity (Brief symptom inventory-18) as the independent variables. The mTBI severity term was not significant.

For mTBI patients and comparisons, we performed a regression analysis with the DCCS score as the dependent variable and [C17dm – C15s] and the group membership variable (comparisons and mTBI) as independent variables. The DCCS score was significantly associated with [C17dm – C15s] (*p*-value = 0.013) and the group membership variable (*p*-value = 0.0207). Then we added an interaction term ([C17dm – C15s] × group membership) to the regression model. This interaction term ([C17dm – C15s] × group membership) was not significant (*p*-value = 0.325).

DCCS is one task of the NIH Toolbox cognition measures which have seven tasks. All of them were administrated. The *p*-value of the association between DCCS and [C17dm – C15s] was not adjusted for multiple tasks (seven tasks in the NIH Toolbox cognition measures).

### Graph Analysis

The nodes in our graph were components in the default mode, salience, and central executive networks. The average path length quantifies the ability for information to propagate in parallel. The AUC of the average path length of mTBI patients was significantly higher than that of controls. The mean AUCs were 2.02 (SD 0.50) and 2.29 (SD 0.64) for controls and mTBI patients, respectively. The two-sample *t*-test *p*-value was 0.028. The global clustering coefficient indicates the extent of the local interconnectivity or cliquishness in a graph. We found the AUC of the global clustering coefficient of mTBI patients was not significantly different from that of controls (*p*-value = 0.54).

Because there were two graph descriptors (the average path length and the global clustering coefficient), we conducted multiple comparisons correction using the false discovery rate. The average path length was marginally significant after correction (adjusted *p*-value = 0.056).

### ReHo Analysis

**Figure 6:**
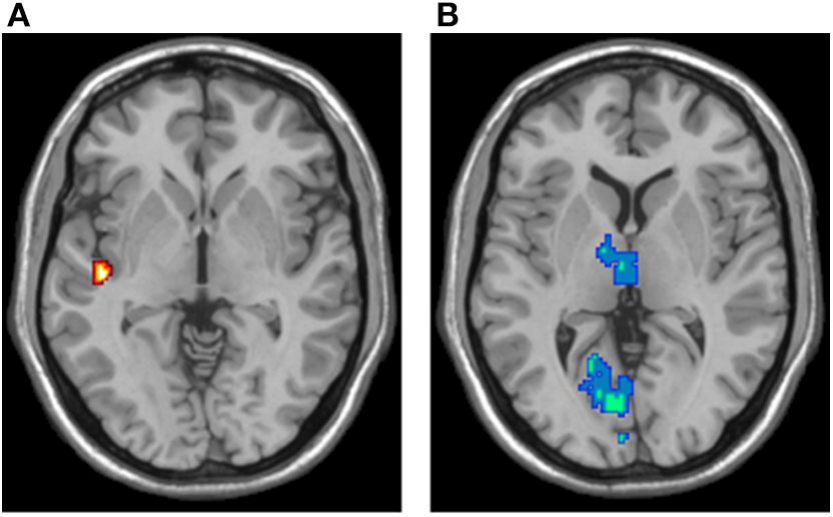
ReHo analysis results, overlaid in the MNI brain. **(A)** Voxels demonstrating significant ReHo increases in the mTBI group (two-sample *t*-test; *p*-value < 0.05, corrected). **(B)** Voxels demonstrating significant ReHo decreases in the mTBI group (two-sample *t*-test; *p*-value < 0.05, corrected).

ReHo results are shown in Figure 6. Compared to controls, mTBI patients showed significant ReHo decreases in the bilateral calcarine fissure and surrounding cortex, left cuneus, left lingual gyrus, and bilateral thalamus. These regions are in the visual and thalamus networks. Compared to controls, mTBI patients showed significant ReHo increases in the left rolandic operculum, left heschl gyrus, and left superior temporal gyrus. These regions are in the auditory network. No voxels in the default mode, salience, and central executive networks showed significant ReHo differences between controls and mTBI patients.

### Anatomical Connectivity Analysis

Using DTI and TBSS, we found no significant anatomical connectivity differences in mTBI patients and comparisons. No voxels in the skeletonized FA space had a corrected *p*-value < 0.05. In ROI-based anatomical connectivity analysis, no ROIs had a corrected *p*-value < 0.05. We found no ROIs that demonstrated significant FA differences in mTBI patients and comparisons.

### Stability Relative to ICA Component Masks

We examined whether our findings were sensitive to the method to generate ICA component masks. Let C2^con^ denote the component mask 2 in the Mask(control) space. We found that C2^con^ matched C2^all^, C9^con^ matched C4^all^, C3^con^ matched C17^all^, C8^con^ matched C8^all^, C11^con^ matched C9^all^ and C20^all^, C17^con^ matched C19^all^, C15^con^ matched C4^all^ and C10^all^.

Our main finding was that [C17dm – C15s] was significantly higher in mTBI patients than controls in the Mask(control) space. We calculated the component coupling in the Mask(all) space which was correspondent to [C17dm – C15s] in the Mask(control) space. This component coupling was still significant (*p*-value = 0.032). The mean functional connectivity scores were 0.179 (SD 0.039) and 0.317 (SD 0.050) for controls and mTBI patients, respectively. The difference in the value of functional connectivity scores in the Mask(control) and Mask(all) spaces may reflect the component mask differences. The finding that [C17dm – C15s] in the Mask(control) space was significantly higher in mTBI patients than controls is not sensitive to the method to generate ICA component masks.

We assessed whether graph analysis results were sensitive to the method to generate ICA component masks [Mask(control) or Mask(all)]. In the Mask(all) space, we found that AUC of the average path length of mTBI patients was still higher than that of controls. The mean AUCs were 1.74 (SD 0.077) and 1.79 (SD 0.087) for controls and mTBI patients, respectively. This pattern was consistent with that in the Mask(control) space. However, the difference was not significant (*p*-value > 0.05). In the Mask(all) space, the AUC of the global clustering coefficient of mTBI patients was not significantly different from that of controls. This was consistent with the finding in the Mask(control) space.

## Discussion

In this study, we examined whether correlations among three intrinsic networks (the default mode, salience, and central executive) were altered in acute mTBI patients. Using component correlation analysis [in the Mask(control) space], we found a pattern of hyper-connectivity between the anterior part of the default mode network and the salience network in acute mTBI patients. This finding was stable across data resampling. For all resampling datasets, we consistently detected this hyper-connectivity pattern; this was a localized pattern. Using graph analysis [in the Mask(control) space] to investigate a graph including nodes in the default mode, salience, and central executive networks, we found a global graph pattern change that indicated altered information propagation ability among nodes; this was a distributed pattern. Together, our study indicated altered couplings among the default mode, salience, and central executive networks in acute mTBI patients.

Our finding of hyper-connectivity between the anterior part of the default mode network and the salience network is consistent with other functional connectivity studies of TBI. In a meta-study of moderate and severe TBI (Hillary et al., 2015), 12 of 14 TBI studies reported hyper-connectivity in different brain regions such as structures in the default mode and salience networks. Shumskaya et al. (2012) analyzed resting-state fMRI data of 35 acute mTBI and 35 age-, gender-, and handedness- matched controls and found a cluster of increased functional connectivity in the right frontoparietal attention network in the mTBI group. In a study to investigate whether thalamic intrinsic connectivity networks are disrupted in patients with mTBI (Tang et al., 2011), Tang et al. analyzed resting-state fMRI data of 24 mTBI patients with mean 22 days post-injury and 17 controls, and found significantly increased thalamic intrinsic connectivity networks in mTBI patients. Hyper-connectivity is also observed in other neurological disorders such as Alzheimer's disease, mild cognitive impairment, and multiple sclerosis (Hillary et al., 2015).

The mechanism underlying hyper-connectivity between the anterior part of the default mode network and the salience network could be a compensatory or maladaptive response (Pievani et al., 2014). In the compensatory theory, hyper-connectivity in acute mTBI is a mechanism to meet cognitive demand. A maladaptive response might reflect an unsuccessful attempt to recruit brain regions to compensate for pathology, as well as a disrupted excitatory-inhibitory balance of damaged networks. We found that the hyper-connectivity in mTBI patients was positively correlated with the DCCS score which measures executive function (Figure 5). This hyper-connectivity predicted better performance in an executive functioning task. This finding suggests that hyper-connectivity between the anterior part of the default mode network and the salience network could be compensatory to meet cognitive demand.

The triple network model centers on the default mode, salience, and central executive networks. The default mode network shows decreased activation in stimulus-driven cognitive and affective information processing tasks, while the salience and central executive networks show increased activation in such tasks. In the triple network model, the salience network is an integral hub in mediating dynamic couplings between the default mode and central executive networks. Inappropriate assignment of saliency to external stimuli or internal mental events is observed in many psychiatric and neurological disorders (Uddin, 2014). In this study, we found the salience network was excessively coupled to the default mode network. This hyper-connectivity may be a response to brain injury to meet cognitive demand.

In our graph analysis, we found that the average path length of mTBI patients was significantly higher than that of healthy subjects. The average path length quantifies the ability for information to propagate. Short path lengths ensure inter-node effective integrity and promote the transfer of information among nodes (Sporns, 2011). Thus, the mTBI-related increase in the average path length represents a distributed and global degeneration of functional connectivity among nodes in the default mode, salience, and central executive networks.

In our ReHo analysis, we found no significant ReHo differences in mTBI patients in voxels in the default mode, salience, and central executive networks. Therefore, the observed hyper-connectivity between the default mode and salience network may not reflect the local functional connectivity changes in the three intrinsic networks.

In our study, no significant anatomical connectivity differences were detected in mTBI patients. Acute mTBI was not associated with DTI-based anatomical connectivity abnormalities detectable with TBSS or ROI-based analysis. This suggests that the observed hyper-connectivity between the default mode and salience network may not reflect the anatomical connectivity changes in acute mTBI patients. Our finding is in accordance with (Ilvesmaki et al., 2014) which analyzed DTI data from 75 patients with acute mTBI and 40 age- and gender- matched controls. Using TBSS, they found no significant differences in FA between mTBI patients and controls. However, TBSS has limitations in that it is a univariate analysis method and cannot model tract couplings. It is possible that the effect of injury on a specific white matter tract is weak. Therefore, TBSS will detect no FA differences between mTBI patients and controls. Using multivariate analysis, the couplings among white matter tracts may provide complementary information about white matter integrity.

Our study used resting-state fMRI and ICA to examine correlations among the default mode, salience, and central executive networks. A related study Jilka et al. (2014) analyzed fMRI data of the Stop Signal Task (SST) of 44 moderate/severe and 13 mTBI and 25 controls. Jilka and colleagues found that for controls, functional connectivity between the salience network and the default mode network transiently increased during stopping; and this change in functional connectivity was not observed in traumatic brain injury patients with impaired cognitive control. Their study revealed abnormal coupling between the salience and default mode networks in traumatic brain injury. The major differences between Jilka's study and our study are: (1) Jilka's study examined the functional connectivity using task-based fMRI while our study used resting-state fMRI; (2) the patient population of Jilka's study was primarily moderate/severe traumatic brain injury while our study focused on mTBI.

This study has several limitations. First, our study is cross-sectional. Investigating brain functional and structural changes in acute mTBI patients is important for predicting prognosis and treatment optimization. Brain connectivity damages in the acute period can result in deterioration of cognitive function that may persist for years. Predicting outcomes based on baseline imaging features is an important problem (Chen and Herskovits, 2015). Future work using a longitudinal design and predictive modeling can address this problem. Second, our study is hypothesis-driven and examines correlations among the default mode, salience, and central executive networks in acute mTBI patients. We cannot exclude the possibility that other network correlations are also changed in mTBI. For example, we didn't examine correlations among the visual and thalamus networks and other networks, despite the visual and thalamus networks showed aberrant ReHo patterns. With the increasing number of intrinsic connectivity networks, we need a large sample size in order to achieve a statistical power to reveal changes in their correlations. We plan to conduct a large-scale network correlation analysis with a large sample size mTBI dataset.

In conclusion, we identified aberrant functional coupling between the default mode and salience networks in acute mTBI patients. Our finding has great potential to improve our understanding of the network architecture of mTBI, leading to accurate diagnosis and more effective treatments.

## Data Availability Statement

The datasets generated are available from the corresponding author on reasonable request.

## Ethics Statement

The studies involving human participants were reviewed and approved by the Institutional Review Board of Shanghai East Hospital. The patients/participants provided their written informed consent to participate in this study.

## Author Contributions

ZW and RC: design and conduct of the study. ZW, RC, YL, XC, WW, MW, GH, and GZ: collection, management, analysis and interpretation of the data, and preparation, review, or approval of the manuscript. All authors: contributed to the article and approved the submitted version.

## Conflict of Interest

The authors declare that the research was conducted in the absence of any commercial or financial relationships that could be construed as a potential conflict of interest.

## Abbreviations

AUC: Area under the curve
BOLD: blood oxygen level-dependent
BSI-18: Brief Symptom Inventory-18
dACC: dorsal anterior cingulated cortex
dlPFC: dorsolateral prefrontal cortex
FA: fractional anisotropy
DTI: diffusion tensor imaging
FIC: frontoinsular cortex
fMRI: functional magnetic resonance imaging
GCS: Glasgow Coma Scale
mPFC: medial prefrontal cortex
mTBI: mild traumatic brain injury
PCC: posterior cingulate cortex
ReHo: regional homogeneity
ROI: region of interest
RPQ: Rivermead Post-Concussion Questionnaire
TBSS: tract-based spatial statistics.
